# Supplementary material for: Vaccination with Plasmids Encoding the Fusion Proteins D-S1, D-S1N and O-SN from SARS-CoV-2 Induces an Effective Humoral and Cellular Immune Response in Mice
Source: Vaccines (Basel). 2025 Jan 28;13(2):134. doi: 10.3390/vaccines13020134 (PMC11860763; doi:10.3390/vaccines13020134)
Supplement: Supplementary file 1 [file vaccines-13-00134-s001.zip › vaccines-3392550-supplementary.pdf]

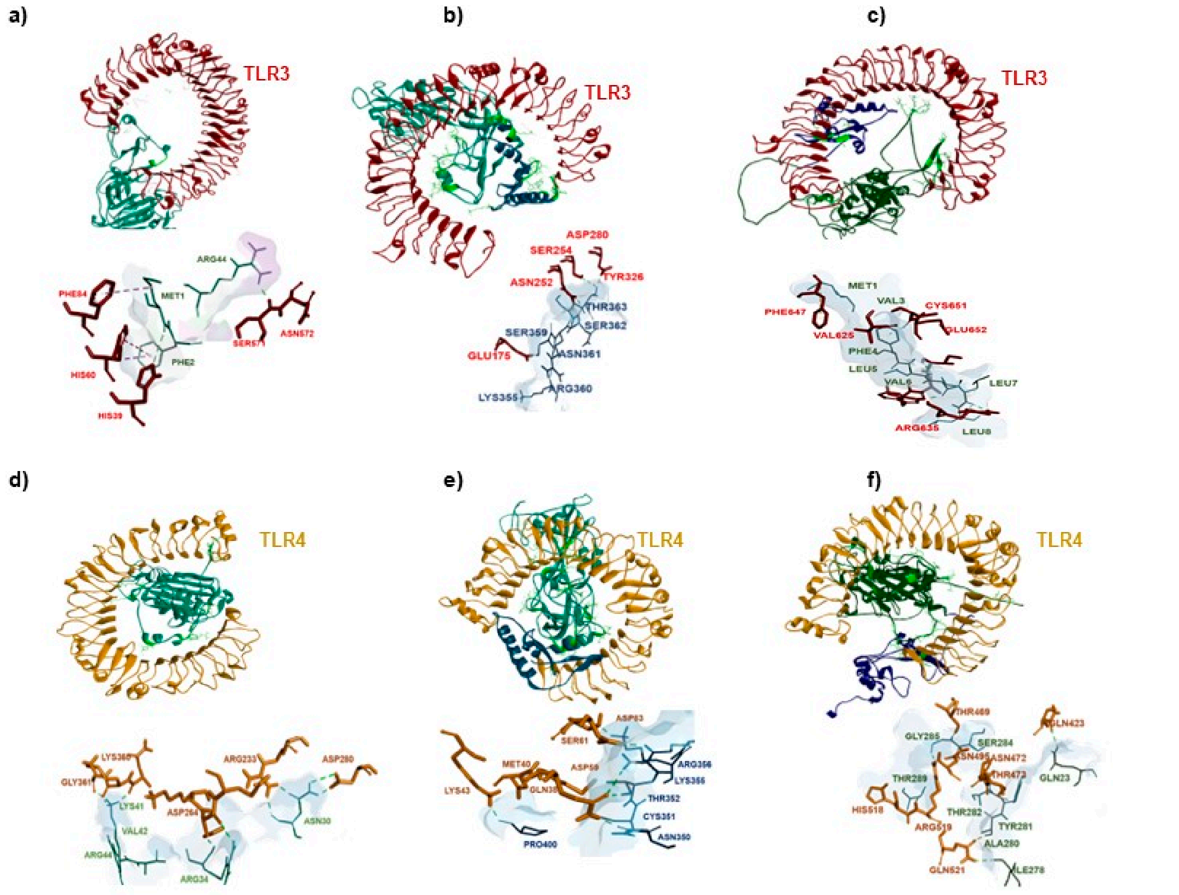

Figure S1: Molecular docking between fusion proteins and TLR3/TLR4

Supplementary Table S1: Amino acids involve in the interaction between fusion proteins and TLR3				
	Fusion protein	TLR3	Bound	Energy Value
D-S1	MET1, PHE2, ARG44, SER45, SER46, VAL47, ASN340, ARG357	SER38, SER39, LEU40, THR59, HIS60, ASN61, GLY83, PHE84, GLN107, HIS108, GLU570, SER571, ASN572, GLY594, LEU595	Hydrogen Electrostatic interactions	-1019.9
D-S1N	MET1, PHE2, GLN14, CYS15, GLN23, PRO26, ASN30, ASP40, LYS41, VAL42, ARG44, SER45, SER46, LEU48, LYS355, SER359, ARG360, ASN361, SER362, PRO400, SER401, GLY402, THR403, TRP404, LYS412, LEU413, TYR434, PHE437, PRO438, PRO439, THR440, GLU441	MET1, PHE2, GLN14, CYS15, GLN23, PRO26, ASN30, ASP40, LYS41, VAL42, ARG44, SER45, SER46, LEU48, LYS355, SER359, ARG360, ASN361, SER362, PRO400, SER401, GLY402, THR403, TRP404, LYS412, LEU413, TYR434, PHE437, PRO438, PRO439, THR440, GLU441	Hydrogen Electrostatic interactions	-1054.1
O-SN	MET1, PHE2, VAL3, PHE4, LEU5, VAL6, LEU7, LEU8, ARG31, VAL33, TYR35, GLY38, SER39, SER41, ARG43, LYS265, HIS267, ARG268, ARG269, ARG440, MET443, GLU444.	HIS32, GLU33, VAL34, THR78, SER79, ASP81, LYS102, VAL103, GLU127, LYS200, ARG251, ASN252, THR277, ASP280, GLU301, TYR302, PHE304, GLU306, GLU358, GLN483, ARG506, LYS531, HIS563, VAL625, ARG635, PHE647, CYS651, GLU652, ILE654, ALA655, TRP656, VAL658, TRP660.	Hydrogen Electrostatic interactions	-1256

Supplementary Table S2: Amino acids involve in the interaction between fusion proteins and TLR4				
	Fusion protein	TLR4	Bound	Energy Value
D-S1	PHE4, LEU10, SER12, GLN14, ASN30, ARG34, LYS41, VAL42, ARG44,	VAL31, VAL32, THR36, GLN38, MET40, ASP41, PHE62, ASP83, GLU128, ASP208, ARG233, LYS263, ASP264, GLU265, ASN359, LYS360, GLY361, ARG380, ASN407, HIS429	Hydrogen Electrostatic interactions	-1343.7
D-S1N	MET1, VAL3, PHE4, VAL6, GLN14, CYS15, ASN17, ARG44, SER46, LEU48, THR57, GLU58, LYS112, CYS113, TYR114, VAL116, SER117, LYS120, ASP162, ASN270, LYS271, ARG301, ILE303, ASP305, ASN350, CYS351, THR352, LYS355, ARG356, SER362, THR363, PRO364, ALA379, PRO400,	GLY38, MET40, LYS43, ASN57, ASP59, SER61, GLN80, TRP81, ASP83, GLU128, LYS152, LYS153, ASN155, SER182, TYR183, LYS262, ASP264, ARG337, MET358, ASN359, LYS360, GLY361, ARG380, ARG400, PHE406, GLU421, GLN423, HIS429, SER430, GLU445, LYS446, TYR454, ASN456, ASN492, THR494, ARG519, GLN521, SER545, PHE570	Hydrogen Electrostatic interactions	-1368.1
OSN	VAL3, LEU5, VAL6, LEU8, GLN23, THR26, SER28, ARG31, TYR34, THR47, GLU48, SER49, LYS252, GLY257, SER259, GLN261, THR262, THR264, ARG269, ARG271, ILE278, ALA280, TYR281, THR282, SER284, GLY285, THR289, ARG440	GLU30, VAL31, GLN38, SER53, ASP59, ASN75, GLU78, TRP81, ASP83, GLU128, LYS153, ALA168, SER171, HIS178, GLN202, GLU229, ARG256, ARG288, ILE336, ARG337, THR355, TYR375, ASP377, ARG380, HIS401, GLN423, LEU448, THR469, ASN472, THR473, ASN495, HIS518, ARG519, GLN521	Hydrogen Electrostatic interactions	-1244.1
